# Comparative Analysis between Ecotoxicity of Nitrogen-, Phosphorus-, and Potassium-Based Fertilizers and Their Active Ingredients

**DOI:** 10.3390/toxics5010002

**Published:** 2016-12-27

**Authors:** Nathan de Castro Soares Simplício, Daphne Heloísa de Freitas Muniz, Fernanda Regina Moreira Rocha, Denis Cavalcanti Martins, Zélia Malena Barreira Dias, Bruno Pereira da Costa Farias, Eduardo Cyrino Oliveira-Filho

**Affiliations:** 1 Embrapa Cerrados, Rodovia BR 020, Km 18, Planaltina DF 73310-970, Brazil; nathan.simplicio@globo.com (N.d.C.S.S.); daphne.muniz@embrapa.br (D.H.d.F.M.); 2 Faculdade de Ciências da Educação e Saúde (FACES), Centro Universitário de Brasília (UniCEUB), Brasília DF 70790-075, Brazil; fernandarmr9@gmail.com (F.R.M.R.); deniscavm@gmail.com (D.C.M.); bruno.faria85@gmail.com (B.P.d.C.F.); 3 Faculdade UnB Planaltina, Campus de Planaltina Área Universitária 01, Universidade de Brasília (UnB), Planaltina DF 73345-010, Brazil; zelia.malena@hotmail.com

**Keywords:** bioassays, agriculture, lethal concentration, environmental chemistry

## Abstract

This study aimed to analyze the ecotoxicity of nitrogen-, phosphorus-, and potassium-based compounds to organisms of two different trophic levels in order to compare the toxic effect between high-purity substances and these substances as components of fertilizers. Dilutions were made with the fertilizers’ potassium chloride, potassium nitrate, superphosphate, urea, and their equivalent reagents, to conduct assays to establish the acute lethal concentration for half of the population (LC50). Ten individuals of the benthic snail *Biomphalaria glabrata* and the fish *Danio rerio* were exposed to each concentration of tested compounds. As a result, the toxicity levels of potassium chloride, potassium nitrate, and urea were obtained for *B. glabrata* and *D. rerio*, with the fish being more susceptible to potassium chloride in the fertilizer and the snail to potassium nitrate and urea, in both commercial and reagent forms. Regarding superphosphate, no significant toxicity was found. This study concluded that among the tested substances, KNO_3_ and KCl were the most toxic substances and urea the least toxic. It was not possible to establish the most sensitive species since, for KCl, the fish were more susceptible to the fertilizer and the snail to the reagent, while for KNO_3_ the opposite was observed.

## 1. Introduction

Within the agricultural production chain, nitrogen (N), potassium (K), and phosphorus (P) are of great importance for the practice of agriculture. In the production process it is difficult for the farmer to obtain a high yield without using at least phosphate-, nitrogen-, and potassium-based fertilizers. According to the International Fertilizer Industry Association [1], on average 104,252 million tons of nitrogen, 40,522 million tons of phosphate, and 27,435 million tons of potassium are used each season worldwide.

Nitrogen, when in aqueous medium, is converted into ammonia, nitrite, and nitrate in a three-step process [2]. It is known that of these three ions, ammonia is the most toxic to aquatic organisms [3], but these compounds often exist at low concentrations in aquatic ecosystems because they are the main source of nitrogen for primary producers [4]. The ecological imbalances and the risks for human consumption are now considered when nitrate and nitrite concentrations become high, due to nutrients deposited in the water body through activities such as agriculture and livestock [3].

Phosphorus can occur in natural and waste waters almost entirely in the form of phosphates. These are classified into orthophosphates, organic phosphates, and condensed phosphates (pyrometaphosphate and other polyphosphates). The third of these is not very relevant to water quality control studies, because condensed phosphates hydrolyze, quickly becoming orthophosphates in natural waters [2]. Orthophosphates are represented by the radicals PO_4_^3−^, HPO_4_^2−^, H_2_PO_4_^−^, and if combined with cations, these form inorganic salts in the water. The discharge of phosphates from raw or treated sewage, agricultural drainage, or certain industrial waste can stimulate the growth of micro and macro photosynthetic organisms in large amounts, triggering the eutrophication process [2].

Potassium (K) is one of the elements most needed by crops; however, its fixation in the soil is more difficult since its salts have low cation exchange capacity [5,6]. This compound is usually extracted from marine sedimentary deposit sources in the form of KCl or potassium sulfate (K_2_SO_4_) [7].

K is also involved in the salinization of the water process, and in some places this salinization can change the composition of local fauna [8]. Due to this effect on salinity, potassium acts indirectly in the toxicity even when present in small concentrations [9].

In the presence of water, many compounds are biochemically interconverted into ions [2]. Other substances present in these compounds, such as impurities, ions, metals, organic contaminants, and even microorganisms, will interact with elements in the environment [10]. When they are transported to a water source, such as lakes, streams, and ponds, the compounds generally undergo alterations in quantity, be it dilution, reconcentration, or transfer. They also undergo alterations in their quality, being changed by degradation processes or multiple reactions that may act during the transport process, forming compounds with distinct properties; in turn, these may even increase or decrease the toxicity of the compound [11].

Works that deal with the action of these ions alone are important in the literature, since the interaction of the elements in the environment can induce toxicity in others [12], as occurs with potassium. It has been observed that when in ionic form, potassium compounds raise the toxicity of other chemicals, such as nitrate [9,10], and that the different forms of soluble nitrogen can define their impact on the environment [13,14,15]. Another study showed that, when in an aqueous solution, cadmium has its toxic effect maximized, depending upon the calcium concentrations in the water [16]. It has also been observed that diluted calcium can reduce the toxicity of phosphate by promoting its precipitation [17,18].

The choice was made to use a chemical reagent and a commercial fertilizer in this study after the authors reviewed articles on the ecotoxicology of compounds used as fertilizers. Those that reported the origin of the element used showed a preference for chemical reagents instead of commercial fertilizers. This fact has some implications within the results analysis, because most of the commercialized fertilizers do not possess the purity that chemical reagents present. Indeed, the purity of potassium in a commercial fertilizer can range from 18% to 60% depending on the fertilizer and the manufacturer [19]. This range makes it difficult to understand if the toxicity is related to the test substance or to impurities contained in the commercial product formulation.

The study aimed to analyze the toxicity of nitrogen-, phosphorus-, and potassium-based compounds in different forms in two organisms from different trophic levels in order to compare the toxic effect between high-purity substances and those used as a component of fertilizer, seeking to identify the element present in the compound responsible for toxicity and the increase or reduction in toxicity.

## 2. Materials and Methods

The species used in this study were the benthic snail (*Biomphalaria glabrata)* and the zebrafish (*Danio rerio*), involving species from two different trophic levels, the first one being a mollusk and the second a fish. Both are freshwater organisms, aiming to cover organisms with distinct biological characteristics and habits, since the snail is a benthic organism and the fish is pelagic. Many studies have already been carried out with planktonic organisms, but few with benthic organisms and, thus, the need to evaluate a model of this trophic level. Fish were included because they are key organisms in aquatic environments, and to be able to obtain a comparative susceptibility with the snails.

The main fertilizers used in the Brazilian Cerrado (high savanna) were used for the study, namely potassium chloride (KCl), potassium nitrate (KNO_3_), single superphosphate (Ca(H_2_PO_4_)_2_ + CaSO_4_), and urea (CO(NH_2_)_2_). From this choice, dilutions were made of the respective commercial fertilizers (CF), and their equivalent chemical reagents (CR) were used as active ingredients, namely, potassium chloride, J.T. Baker^®^ (purity 99.8%) (J.T. Baker^®^, Xalostoc, Mexico), potassium nitrate, Impex^®^ (purity 99%) (Impex, RS, Brazil), urea, Merck^®^ (purity 100%) (Merck^®^, Darmstadt, Germany) and potassium phosphate monobasic, Merck^®^ (purity 99.5%) (Merck^®^, Darmstadt, Germany); the latter was used for comparison with superphosphate.

The dilution concentrations ranged from 75 to 3000 mg∙L^−1^ for the CR and 100 to 3000 mg∙L^−1^ for the CF. For urea, which is known to trigger mortality at higher concentrations, dilutions ranged from 1000–40,000 mg∙L^−1^ for the CR and 10,000–40,000 mg∙L^−1^ for the CF. All of these concentrations were defined based on preliminary tests. The concentrations used in the definitive tests were variable for each CR, being obtained from preliminary tests with about three well-spaced dilutions.

After dilutions, acute assays in laboratory with the chosen organisms were performed to establish the lethal concentration for 50% of the studied population (LC50). The acute test method for snails was proposed by Oliveira-Filho et al. [20], and for fish the protocol used was standardized by guideline NBR 15088 of the Brazilian Association for Technical Standardization [21]. For all organisms, LC50s and their respective 95% confidence intervals were determined by the Trimmed Spearman Karber program [22] version 1.5.

In tests with snails the static exposure system was used, and the solutions were prepared only at the beginning of the test, without renewal until the end of exposure. For urea, in particular, and for all tests with fish, the semi-static methodology was used, where solutions were renewed daily to avoid losses by volatilization and degradation, as recommended by guideline EPA 821-R-02-012 [23]. During the test, pH and dissolved oxygen were checked daily with the intention of identifying possible abrupt changes in those parameters during the exposure period.

Before each test, solutions were placed in 2 L beakers for the snails and 3 L for the fish and stirred for 24 h in an orbital shaker. After that, 10 organisms were inserted into each concentration, with the presence of a control group, and the mortality of individuals after 48 and 96 h of exposure was assessed.

The preparation of the dilution water (reconstituted soft water) for all of the tests followed the standardized protocols under guideline NBR 15088 [21], which involved dissolving 48 mg of sodium bicarbonate (NaHCO_3_), 30 mg of calcium sulfate (CaSO_4_), 30 mg of magnesium sulfate (MgSO_4_), and 2 mg of magnesium sulfate (KCl) in 1 L of distilled water. The pH of the dilution water was maintained at 7.2 ± 0.1 and hardness between 40–48 mg∙L^−1^ as CaCO_3_.

All applicable international, national, and institutional guidelines for the care and use of animals were followed. All tests with *Danio rerio* were approved by the Animal Use Commission from the Brasília University Centre—UniCEUB—(file number 003/15), on 19 March 2015.

To ensure the concentrations of the diluted chemicals in the assays, and to define the effective concentrations to be used in the calculation of the LC50, ion chromatography analysis of each sample was performed before and after the assays, including the water destined for the control. Presence of the major component ions ammonium (NH_4_^+^), potassium (K^+^), chlorine (Cl^−^), nitrate (NO_3_^−^), and phosphate (PO_4_³^−^) was analyzed. 

The equipment used for this purpose was the Ionic Chromatograph 761 Compact IC, Metrohm. Ion chromatography is a chromatographic technique that applies the principles of ion exchange, so that electrical conductivity is used for the detection and quantitative determination of ions in solution [24].

To guarantee quality assurance and quality control of determinations, calibration standards were prepared each day of analysis, using Merck^®^ (Darmstadt, Germany) reagents. Blanks, duplicates, and spiked samples were also used. Calibration coefficients had to maintain at least three nines before proceeding with samples (r = 0.999).

To ascertain if there were any significant differences in nutrient concentrations before and after each test, samples were analyzed before and after the exposure time, and the results obtained from ion chromatography were subjected to statistical analysis. We performed the Shapiro-Wilk normality test with significance level of 5% (“shapiro.test” function) to define the statistical test to be used. For data with normal distribution, we performed the paired t-test (“t.test” function), while for those that did not obtain normal distribution we performed the Wilcoxon-Mann-Whitney test (“wilcox.test” function). 

These statistical procedures were performed with the R software package, 2.15.3 version [25] and had the intention of examining only if there was a significant change in the analyzed nutrient concentrations during the exposure period. 

## 3. Results

In all tests the analysis of ion chromatography showed, as expected, the presence of chloride (Cl^−^) and potassium (K^+^) in samples destined for the control group and in the dilution water at concentrations of 0.649 mg∙L^−1^ for Cl^−^ and 2.079 mg∙L^−1^ for K^+^, as is expected for the use of KCl in the preparation of the dilution water.

The urea concentrations employed in the experiments had a high variation in chromatographic analyses, a very dynamic degradation when diluted, so when analyzed at some concentrations, the substance already showed a stage that could not be detected.

The Shapiro-Wilk normality test did not show normal distribution for the magnesium variable in KCl dilutions. For the KNO_3_ dilutions, the normality test did not show normal distribution either for the potassium or the nitrate. In both cases the sample size was 10. For urea dilutions, the normality test revealed a normal distribution for all of the variables. Finally, for potassium phosphate monobasic and superphosphate, the normality test also showed a normal distribution for all of the variables. 

However, neither the T test nor the Wilcoxon-Mann-Whitney test (for the variable not normally distributed) showed a significant difference between the initial dilution and the final one. As the statistical test showed no significant difference between the chemical analysis performed before and after the tests, in all subsequent tables only the results of analysis before the tests are shown, aiming for a better presentation of the data (Table 1 and Table 2).

**Figure 1:**
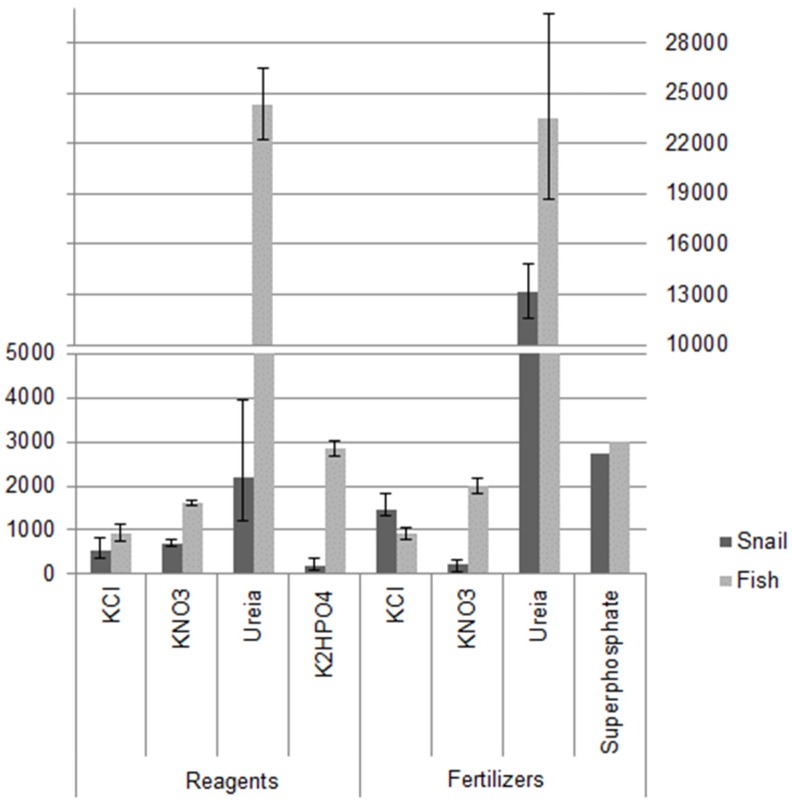
Lethal concentrations (LC50s) found for *B. glabrata* snail and *D. rerio* fish within 96 h of exposure (units: mg∙L^−1^). Error bars represent 95% confidence intervals.

After the end of all assays it was possible to determine which was the most toxic substance and which organism was most sensitive to the tested fertilizer within 96 h of exposure (Figure 1).

In the experiments involving the species *B. glabrata* and the calculated LC50 above, it is possible to see the difference in toxicity of the CR compared with that of the CF in all tested substances. The CR was more toxic than the CF in practically all cases, except for KNO_3_, where the opposite was observed. For *D. rerio*, only KNO_3_ showed a statistical difference in the toxicity of the CR in relation to the CF. No other compound showed a difference because of the confidence interval limits.

From chemical analysis we performed the calculation of toxicity from each of the substances, which can be seen in the tables below.

Table 3 shows that potassium toxicity was affected by the presence of other elements in the tested dilutions, since the potassium toxicity varied individually in function of the substance tested and the exposed organism. It was shown that K^+^ in KH_2_PO_4_ was more toxic than K^+^ in KCl and KNO_3_ (and there was no significant difference between them) for *B. glabrata*. However, for *D. rerio*, K^+^ in KCl was more toxic than the one in KNO_3_ and KH_2_PO_4_ (and there were no significant differences between them).

Table 4, in turn, shows that nitrogen toxicity can be maximized because of its dissociation in water, since the compound as NH_4_^+^ was showed to be more toxic than its form as NO_3_^−^. Moreover, the greater toxicity of N for *B. glabrata* can be associated with a higher intolerance of the species to the compound in relation to time, since the values at 96 h diverge from the others. 

When analyzing ammonium toxicity to the snail, low LC50 values were observed, reaching close to zero for the CR. This shows great sensitivity of the species to nitrogen in NH_4_^+^ form. When the toxicity of ammonium in the urea compound for the fish is analyzed, it is noted that this has already raised mortality in very small concentrations, with LC50 under 20 mg∙L^−1^ being observed for all organisms. It is also possible to see more NH_4_^+^ toxicity contained in the CR for *B. glabrata* in 48 h of exposure, but with no difference between both species when only results at 96 h are evaluated due to the large confidence interval.

For the phosphate anion, Table 5 showed that the toxicity was only compared between species, with *B. glabrata* being shown to be more sensitive to this element than *D. rerio*. These results, together with those obtained with K^+^ as the donor source for the substance, showed that this ion is more responsible for the toxicity found for the fish than the PO_4_^3−^ for potassium phosphate monobasic substance.

## 4. Discussion

When the toxicity data found for snails and for fish were compared, there was greater sensitivity of fish than snails to the CF KCl, unlike what was found for the CR. 

However, when the toxicity found for KCl was compared with other species (Table 6), it was noted that in relation to the CR (Figure 1) no difference was found in KCl toxicity in similar tests at 48 h. When compared with those obtained for KCl in CF (Figure 1), *B. glabrata* stands out from the others as the second-most resistant, behind only the insect *C. triangulifer*.

The toxicity data obtained for KNO_3_ showed higher sensitivity to the substance from *B. glabrata* than *D. rerio*, both for the CR and for the CF (Figure 1), but the snail also showed higher sensitivity to the CF, while the fish showed higher sensitivity to the CR. The results of the toxicity tests for KNO_3_ in this study showed less resistance among the organisms used than for others in the literature (Table 7).

When results were compared both with the CR and with the CF KNO_3_, all results showed the snail to be the second-most sensitive of all compared species, after the cladoceran. However, when the test results obtained with CF urea were compared, it was not possible to detect which species was more sensitive to fertilizer because of the confidence interval limits of 48 h of exposure. However, with the 96-h results, the snail was more sensitive than the zebrafish to the CF. 

The data of this study compared with similar ones in the literature show that urea is also toxic to other species only at high concentrations (Table 8).

All results presented in these studies, when compared with the results for the organisms used in the present study (Figure 1), indicate that *B. glabrata* is the most sensitive to urea as a reagent. However, if only the CF is analyzed, the species *C. catla* appears to be the most sensitive, followed by *R. sylvatica*, *B. glabrata*, and *D. rerio* together.

The higher sensitivity of *B. glabrata* to urea can be explained by the fact that this substance has a molluscicidal effect [35], but although urea has this effect on snails, *B. glabrata* was more sensitive than the freshwater snails used by Tchounwou et al. [31]. This may be related to the type of bioassay that was used, because in this study there was daily renewal of test solutions, and in the work of these authors the exposure methodology was not explained. 

From the results in Table 9 it could be seen that the snail was the most sensitive species for the superphosphate at 96 h exposure, but in the 48-h test it was not possible to define which species is most sensitive to fertilizer. The most susceptible species to KH_2_PO_4_ was also *B. glabrata* based on data found in Figure 1.

The results found in the literature for KH_2_PO_4_ show greater resistance of zebrafish over other species. In a study involving the zebra mussel (*Dreissena polymorpha*) (LC50 = 92 mg∙L^−1^) and the Asian clam (*Corbicula fluminea*), Fisher et al. [17] found very different toxicity values for these closely related organisms, making it impossible to find mortality for the species *C. fluminea* in concentrations up to 2000 mg∙L^−1^ of KH_2_PO_4_ in 24 h. In addition to this author, Reish [36], working with the tolerance of polychaete *Nereis grubei*, *Neanthes arenaceodentata*, *Dorvillea articulate*, and *Capitella capitata* to the substance KH_2_PO_4_, obtained mean values of tolerance equal to 0.920 mg∙L^−1^, 1.900 mg∙L^−1^, 2.100 mg∙L^−1^, and 2.400 mg∙L^−1^, respectively, for the test-species.

For CF, the toxicity observed for snails only at a concentration of 3000 mg∙L^−1^ superphosphate can be explained from the moment that Vieira and Ramos [37] report that when the calcium concentration in water is above 120 mg∙L^−1^, phosphate fertilizers tend to precipitate. Therefore, it is possible to assume that the presence of calcium in the dilution water, plus calcium present in high concentrations of superphosphate promoted, the precipitation of phosphate in water. Since *B. glabrata* has a benthic habit, its mortality may be because of the decantation of the substance. Moreover, even if the presence of calcium in water is beneficial for the formation of the snail shell, *B. glabrata* can only tolerate concentrations of up to 120 mg∙L^−1^ calcium [38]. 

In a study which also aimed to observe phosphate toxicity to aquatic organisms of three different trophic levels (fish, algae, and cladocerans), involving the compounds tricalcium phosphate (Ca_3_(PO_4_)_2_) and calcium hydrogenorthophosphate (CaHPO_4_), Kim et al. [18] could not specifically stipulate phosphate toxicity to the organisms tested, because the low solubility of the phosphate prevents assays from being performed in concentrations above 100 mg∙L^−1^. Both in our study and in the research of Kim et al. [18] it can be seen that there is a difficulty in determining phosphate toxicity to aquatic organisms due to the low solubility of this compound and its tendency to precipitate in the presence of calcium.

From the data found for the snail and those listed in Table 9, it is possible to estimate that the toxicity to the fish should be above 4000 mg∙L^−1^ superphosphate, since Omoregie et al. [39] working with Nile tilapia (*Oreochromis niloticus*) found a similar value.

The toxicological data of the individual elements in the tests with the CR (Table 3) showed that *B. glabrata* tends to be more resistant to potassium separately. Indeed, the Brazilian Ministry of Health publication on the control of mollusks [42] shows that this species is resistant to concentrations between 0.1 to 54.5 mg∙L^−1^ of potassium, and in this study all of the calculated LC50s for *B. glabrata*, and their confidence interval limits, were above this value.

When comparing the toxicity values obtained in this study and listed in Table 3 with others in the literature, the tested snails showed greater LC50 for potassium in the 48-h test than in that performed by Freitas and Rocha [43] with the cladoceran *Pseudosida ramosa* (LC50 = 17.7 mg∙L^−1^). However, all of these results were lower than *D. rerio* for KCl, KNO_3_, and KH_2_PO_4_, and the results obtained by Vijayavel and Balasubramanian [44] for the Mozambique tilapia (*Oreochromis mossambicus*) in the 96-h test (LC50 = 569.25 mg∙L^−1^). This showed that in a potassium tolerance range, *P. ramosa* was the most sensitive, followed by *B. glabrata,* and the most resistant were the fish *D. rerio* and *O. mossambicus*.

For nitrogen, different forms of this had different toxicity values for the species, with nitrogen in the form of ammonia (NH_3_) or ammonium (NH_4_^+^) being generally more toxic than nitrogen in nitrate form (NO_3_^−^) [3,45,46]. *B. glabrata* and *D. rerio* were demonstrated to be more sensitive than other species to NO_3_^-^ ions when comparing the data contained in Table 4 with tests performed with sodium nitrate (NaNO_3_) in other organisms. As an example, we can mention the test performed by Alonso and Camargo [45] with the aquatic snail *Potamopyrgus antipodarum*, which had LC50 equal to 1042 mg∙L^−1^, while the test conducted by Hamlin [46] with the Siberian sturgeon (*Acipenser baerii*) had toxicity equal to 1028 mg∙L^−1^; all of these tests took place after 96 h of exposure. With the 48 h of exposure results, there was only a difference when compared again with LC50 values for the *A. baerii* (LC50 = 1443 mg∙L^−1^) [46] and the fathead minnow *P. promelas* (LC50 = 1341 mg∙L^−1^), but without a difference for *C. dubia* and *D. magna* Cladocera (LC50 = 374 mg∙L^−1^ and 462 mg∙L^−1^, respectively) found by Scott and Crunkilton [47], due to the limits of the confidence interval. 

When comparing the results with KNO_3_ as donor source of NO_3_^-^ for the tests, *B. glabrata* and *D. rerio* were more resistant when compared with the guppy fish (*P. reticulata*) in the assays performed by Rubin and Elmaraghy [30], where the toxicity was equal to 191 mg∙L^−1^ after 96 h of exposure.

The toxicity data for NH_4_^+^ (Table 4) showed higher sensitivity of *B. glabrata* in relation to the others. The snail *P. antipodarum* in Alonso and Camargo’s test [45] was the second most vulnerable species, with LC50 equal to 2.23 mg∙L^−1^, followed by pacú fish (*P. mesopotamicus*) with LC50 equal to 3.98 mg∙L^−1^ [15], *D. rerio* and guppy (*P. reticulata*), with a toxicity value equal to 93.9 mg∙L^−1^ [30], the latter being the most resistant species to NH_4_^+^ if only the 48-h results are analyzed. Examining the 96-h data, due to the large confidence interval that *B. glabrata* showed, it was not possible to see any difference between this organism and the other, leaving only *P. reticulata* as the most resistant (LC50 equal to 91.2 mg∙L^−1^).

The higher survival of fish for ammonia in relation to other species may be due to the fact that these organisms are more susceptible to the un-ionized form of ammonia (NH_3_) than its ionized form, or ammonium form (NH_4_^+^), since fish membranes are more permeable to NH_3_ than to NH_4_^+^ [13,14]. This fact becomes clear when studies that aimed to compare the toxicity of NH_3_ with NH_4_^+^ in fish are analyzed. The calculated lethal concentrations for NH_3_ within the first 24 h of exposure ranged from 0.023 to 0.85 mg∙L^−1^ in function of exposed fish species [14,15].

The extreme vulnerability of *B. glabrata* to ammonium in urea was not expected, since it is known that this species can survive in concentrations from 0.1 to 2.6 mg∙L^−1^ NH_3_ [42], and when other snail species are exposed to ammonium sources derived from CR (as ammonium sulfate) and CF (as urea), they did not obtain the same mortality as the species in our study. Toxicity values ranging from 490.79 to 700.73 mg∙L^−1^ were reported for ammonium sulfate [31] and from 48.86 to 54.86 mg∙L^−1^ for urea [32].

Due to the lack of studies addressing phosphate toxicity in the available literature, the use of Ecotox database [41] and Pesticide Info database [40] was required to find LC50 values to have a criterion for comparing our study with others. The search criteria were the substances H_3_PO_4_, Na_2_HPO_4_, NaH_2_PO_4_, CaHPO_4_, Ca(H_2_PO_4_)_2_, and Ca_3_(PO_4_)_2_ for being phosphate sources when diluted; only tests that covered the period of 48 and 96 h of exposure were sought.

The toxicity data for PO_4_^3-^ showed that there is some difficulty in defining the toxicity of this ion to aquatic organisms, and probably its interaction with other compounds can interfere in its toxicity, as highlighted by Fisher et al. [17]. Kim et al. [18] could not define the toxicity of PO_4_^3^^−^ for the amphidromous fish *Oryzias latipes* and the cladoceran *D. magna,* using the compounds CaHPO_4_ and Ca_3_(PO_4_)_2_ as phosphate source, but when it interacts with sodium, the phosphate appears to be less toxic than other variations, since toxicity values were found equal to 3580 mg∙L^−1^ for *D. magna* and 720 mg∙L^−1^ for the mosquito-fish fry (*Gambusia affinis*) in 48-h assays. The most toxic form of the phosphate appears to be phosphoric acid (H_3_PO_4_), with toxicity values equal to 60 mg∙L^−1^ and 87 mg∙L^−1^ being found for the freshwater bluegill sunfish (*L. macrochirus*) and the rainbow trout (*Oncorhynchus mykiss*), respectively, in 96-h assays and 138 mg∙L^−1^ for *G. affinis* at 48 h [40,41].

For KH_2_PO_4_ data found in this study (Table 5), *B. glabrata* showed toxicity data inside the confidence limit that other species had to H_3_PO_4_, but *D. rerio* showed higher tolerance to KH_2_PO_4_.

Based on the results of our study for the KH_2_PO_4_ presented in Table 5, and the ones found in the literature, it can be noted that the phosphate present in Na_2_HPO_4_, NaH_2_PO_4_, CaHPO_4_, Ca(H_2_PO_4_)_2_, and Ca_3_(PO_4_)_2_ is less toxic than that present in KH_2_PO_4_ and H_3_PO_4_ when only acute toxicity data are analyzed. However, it is worth noting that data from chronic toxicity to *O. mykiss* shown by Satoh et al. [48] showed the opposite of those found for acute toxicity. In the latter, the mortality was higher in sodium phosphate and potassium phosphate in the long-term, and the survival was higher in phosphoric acid and calcium phosphate, but with growth reduced in the latter one.

It is emphasized that for all of the ecotoxicological tests compared here, the differences in the toxicity observed may not only be related to the substance or the compound itself, but also to the life stage of the organism used in the assay. It is known that younger organisms tend to be more susceptible than adult organisms [23]. Given the results presented here, it can be concluded that the fertilizers KCl, KNO_3_, and urea have toxic effects on *B. glabrata* and *D. rerio*, with the zebrafish being more susceptible to KCl present in fertilizer and the snail to the other compounds. In the present study, it was only possible to establish a difference between the toxicity to the snail of CF and of CR.

## 5. Conclusions

Faced with the data presented, it can be concluded that KCl, KNO_3_, and urea fertilizers have acute toxic effects on *B. glabrata* and *D. rerio*. The fish is more susceptible to KCl as a commercial fertilizer (CF), and the snail to KNO_3_ and to urea, both as a chemical reagent (CR). Regarding the superphosphate, significant toxicity values were not found. 

Within the presented data, it was possible to conclude that among the CRs, the most toxic was potassium phosphate (KH_2_PO_4_) to the snail and potassium chloride (KCl) to the fish; urea was the least toxic for both organisms. Among the commercial fertilizers (CFs), KNO_3_ was more toxic to *Biomphalaria glabrata* snails, and again KCl was more toxic for the fish *Danio rerio*. Once again, urea was the least toxic CF for both organisms.

It was not possible to establish the most sensitive species, since in some cases one species was more susceptible to a CF and another to CR, This was the case for KCI, where the fish was more susceptible to the CF and the snail to the CR, while for KNO_3_ the opposite was observed.

## Figures and Tables

**Table 1:** Ion chromatography of the dilutions from the chemical reagents (units: mg∙L^−1^).

Substance	Concentration	Ammonium	Potassium	Chloride	Nitrate	Phosphate
KCl	Lowest Concentration	0.000	113.404	132.215	0.000	0.000
	Highest Concentration	0.000	1038.781	1043.386	0.000	0.000
KNO3	Lowest Concentration	0.000	105.629	2.553	100.671	0.000
	Highest Concentration	0.000	1343.164	0.000	1314.378	0.000
Urea	Lowest Concentration	0.000	1.255	1.867	0.000	0.000
	Highest Concentration	12.357	0.000	1.669	0.000	0.000
KH2PO4	Lowest Concentration	0.000	22.590	0.240	0.000	66.702
	Highest Concentration	0.000	1001.143	0.000	0.000	2732.077
Dilution water	-	0.000	2.079	0.649	0.000	0.000

**Table 2:** Ion chromatography from the dilutions of the commercial fertilizers (units: mg∙L^−1^).

Substance	Concentration	Ammonium	Potassium	Chloride	Nitrate	Phosphate
KCl	Lowest Concentration	0.000	101.197	128.476	0.000	0.000
	Highest Concentration	0.000	1020.555	854.058	0.000	0.000
KNO3	Lowest Concentration	0.000	38.875	1.442	41.208	0.000
	Highest Concentration	0.000	1055.488	0.000	1135.409	0.000
Urea	Lowest Concentration	2.300	5.263	5.742	0.000	0.000
	Highest Concentration	20.867	0.000	0.000	0.000	0.000
Superphosphate	Lowest Concentration	0.000	0.000	1.495	0.000	28.753
	Highest Concentration	0.000	0.000	0.000	0.000	576.473
Dilution water	-	0.000	2.079	0.649	0.000	0.000

**Table 3:** Comparison between the lethal concentrations (LC50s) obtained in different assays with potassium present for the reagent and the commercial fertilizer for the *B. glabrata* snail and the *D. rerio* fish (units mg∙L^−1^). Values in brackets represent the 95% confidence intervals.

**Potassium in Reagent**	**Chemical Reagent**			
	**48 h**		**96 h**	
	*B. glabrata*	*D. rerio*	*B. glabrata*	*D. rerio*
**KCl – K**	422.27 (270.14–660.07)	802.39 (523.53–1229.78)	277.98 (183.58–420.92)	527.91 (432.64–644.16)
**KNO3 – K**	347.07 (303.49–396.89)	786.45 (732.92–843.89)	347.07 (303.49–396.89)	786.45 (732.92–843.89)
**KH2PO4 – K**	90.32 (33.11–246.34)	811.47 (690.15–954.12)	44.26 (19.83–98.75)	774.16 (721.89–830.21)
**Potassium in Fertilizer**	**Commercial Fertilizer**			
	**48 h**		**96 h**	
	*B. glabrata*	*D. rerio*	*B. glabrata*	*D. rerio*
**KCl – K**	891.98 (801.01–993.29)	589.25 (502.64–690.79)	785.20 (610.17–1010.43)	509.65 (445.97–582.42)
**KNO3 – K**	139.90 (111.63–175.33)	993.65 (944.33–1045.55)	102.73 (68.40–154.29)	993.65 (944.33–1045.55)

**Table 4:** Comparison between the lethal concentrations (LC50s) obtained in different assays with nitrogen present for the reagent and the commercial fertilizer for the *B. glabrata* snail and the *D. rerio* fish (units: mg∙L^−1^). Values in brackets represent 95% confidence intervals.

**Nitrogen in Reagent**	**Chemical Reagent**			
	**48 h**		**96 h**	
	*B. glabrata*	*D. rerio*	*B. glabrata*	*D. rerio*
**KNO_3_ – N**	354.09 (311.55–402.44)	856.23 (825.64–887.95)	354.09 (311.55–402.44)	856.23 (825.64–887.95)
**CO(NH_2_)_2_ – N**	0.81 (0.62–1.05)	9.05 (8.36–9.81)	0.10 (0.001–17.73)	8.55 (8.08 –9.04)
**Nitrogen in Fertilizer**	**Commercial Fertilizer**			
	**48 h**		**96 h**	
	*B. glabrata*	*D. rerio*	*B. glabrata*	*D. rerio*
**KNO_3_ – N**	153.38 (125.98–186.87)	988.01 (878.70–1110.93)	117.40 (79.88–172.56)	988.01 (878.70–1110.93)
**CO(NH_2_)_2_ – N**	8.51 (5.38–13.45)	6.28 (4.65–8.48)	3.39 (2.89–3.99)	6.28 (4.65–8.48)

**Table 5:** Comparison between the lethal concentrations (LC50s) obtained in different assays with phosphate present for the reagent and the commercial fertilizer for the *B. glabrata* snail and the *D. rerio* fish (units: mg∙L^−1^). Values in brackets represent 95% confidence intervals.

Phosphate in Reagent and in Fertilizer	48 h		96 h	
	*B. glabrata*	*D. rerio*	*B. glabrata*	*D. rerio*
**KH_2_PO_4_ – PO_4_**	260.75 (105.14–646.65)	2171.37 (1811.21–2603.15)	133.73 (61.00–293.16)	2056.98 (1901.46–2225.22)
**Ca(H_2_PO_4_)_2_ + CaSO_4_ – PO_4_**	>576.473	>576.473	515.26	>576.473

**Table 6:** Lethal or effective concentration and their confidence intervals (when present, shown in parentheses) for potassium chloride in studies with aquatic organisms.

Species Name	LC50/EC50 (95% Confidence Interval Limit)	Study
*Ceriodaphnia dubia*	630.0 mg∙L^−1^ (580–670)	Mount et al. (1997) [ 26]
*Daphnia magna*	660.0 mg∙L^−1^ (440–880)	Mount et al. (1997) [ 26]
*Daphnia similis*	986.66 mg∙L^−1^ (293.34–1313.32)	Utz and Böhrer (2001) [ 8]
*Pimephales promelas*	910.0 mg∙L^−1^ (750–1090)	Mount et al. (1997) [ 26]
*Piaractus mesopotamicus*	1370 mg∙L^−1^	Ignácio et al. (2014) [ 27]
*Centroptilum triangulifer*	1956.7 mg∙L^−1^	Struewing et al. (2015) [ 28]

**Table 7:** Lethal or effective concentration for potassium nitrate in studies with aquatic organisms.

Species Name	LC50/EC50	Study
*Daphnia magna*	490 mg∙L^−1^	Dowden and Bennet (1965) [29]
*Lepomis macrochirus*	5500 mg∙L^−1^	Dowden and Bennet (1965) [29]
*Poecilia reticulata*	1380 mg∙L^−1^	Rubin and Elmaraghy (1977) [30]

**Table 8:** Lethal or effective concentration and their confidence intervals (when present, shown in parentheses) for urea in studies with aquatic organisms.

Species Name	LC50/EC50 (95% Confidence Intervals)	Study
*Biomphalaria havanensis*	21,412.08 mg∙L^−1^ (19,269.66–23,538.43)	Tchounwou et al. (1991) [31]
*Helisoma trivolvis*	13,476.59 mg∙L^−1^ (11,957.82–14,888.23)	Tchounwou et al. (1991) [31]
*Eobania vermiculata*	54,860 mg∙L^−1^	Eshra (2014) [32]
*Theba pisana*	48,860 mg∙L^−1^	Eshra (2014) [32]
*Catla catla*	280 mg∙L^−1^ (260–280)	Sangeetha et al. (2011) [33]
*Rana sylvatica*	14,370 mg∙L^−1^ (12,400–16,110)	Harless et al. (2011) [34]

**Table 9:** Lethal or effective concentrations and their confidence intervals (when present, shown in parentheses) for potassium phosphate monobasic in studies with aquatic organisms.

Species Name	LC50/EC50 (95% Confidence Interval Limit)	Study
*Oreochromis niloticus*	3760 mg∙L^−1^ (2990–4010)	Omoregie et al. (2009) [39]
*Chironomus yoshimatsui*	1520 mg∙L^−1^	Kegley et al. (2014) [40]
*Viviparus bengalensis*	2280 mg∙L^−1^	Kegley et al. (2014) [40]
*Radix luteola*	3000 mg∙L^−1^	USEPA, (2015) [41]
